# Predicting Recovery of Voluntary Upper Extremity Movement in Subacute Stroke Patients with Severe Upper Extremity Paresis

**DOI:** 10.1371/journal.pone.0126857

**Published:** 2015-05-14

**Authors:** Chia-Lin Koh, Shin-Liang Pan, Jiann-Shing Jeng, Bang-Bin Chen, Yen-Ho Wang, I-Ping Hsueh, Ching-Lin Hsieh

**Affiliations:** 1 School of Occupational Therapy, College of Medicine, National Taiwan University, Taipei, Taiwan; 2 Department of Physical Medicine and Rehabilitation, National Taiwan University Hospital, Taipei, Taiwan; 3 Department of Neurology, National Taiwan University Hospital, Taipei, Taiwan; 4 Department of Medical Imaging and Radiology, National Taiwan University Hospital, Taipei, Taiwan; Emory University School Of Medicine, UNITED STATES

## Abstract

**Background and Objective:**

Prediction of voluntary upper extremity (UE) movement recovery is largely unknown in patients with little voluntary UE movement at admission. The present study aimed to investigate (1) the extent and variation of voluntary UE movement recovery, and (2) the best predictive model of the recovery of voluntary UE movement by clinical variables in patients with severe UE paresis.

**Design:**

Prospective cohort study.

**Methods:**

140 (out of 590) stroke patients with severe UE paresis completed all assessments. Voluntary UE movement was assessed using the UE subscale of the Stroke Rehabilitation Assessment of Movement (STREAM-UE). Two outcome measures, STREAM-UE scores at discharge (DC_STREAM-UE_) and changes between admission and discharge (Δ_STREAM-UE_), were investigated to represent the final states and improvement of the recovery of voluntary UE movement. Stepwise regression analyses were used to investigate 19 clinical variables and to find the best predictive models of the two outcome measures.

**Results:**

The participants showed wide variation in both DC_STREAM-UE_ and Δ_STREAM-UE_. 3.6% of the participants almost fully recovered at discharge (DC_STREAM-UE_ > 15). A large improvement (Δ_STREAM-UE_ >= 10) occurred in 16.4% of the participants, while 32.9% of the participants did not have any improvement. The four predictors for the DC_STREAM-UE_ (R^2^ = 35.0%) were ‘baseline STREAM-UE score’, ‘hemorrhagic stroke’, ‘baseline National Institutes of Health Stroke Scale (NIHSS) score’, and ‘cortical lesion excluding primary motor cortex’. The three predictors for the Δ_STREAM-UE_ (R^2^ = 22.0%) were ‘hemorrhagic stroke’, ‘baseline NIHSS score’, and ‘cortical lesion excluding primary motor cortex’.

**Conclusions:**

Recovery of voluntary UE movement varied widely in patients with severe UE paresis after stroke. The predictive power of clinical variables was poor. Both results indicate the complex nature of voluntary UE movement recovery in patients with severe UE paresis after stroke.

## Introduction

Severe upper extremity (UE) paresis seriously impacts the quality of life of patients with stroke [1]. Patients with severe UE paresis may have no or very limited voluntary UE movement [2]. They may perform partial UE movement, usually scapular, shoulder, or elbow movement, but they are unable to do simple daily tasks (e.g., hold a cup) [1]. Although only about one-third of patients suffer from severe UE paresis after stroke [2], they consume the majority of medical and social resources [1,3]. Research has shown that the severity of one’s impairment in voluntary control of UE motor units, paresis, is the primary determinant of UE functional loss and daily function restriction after stroke [4]. The voluntary UE movement was found to recover rapidly during the first three to six months and then to slow down in the chronic phase of recovery [5]. Therefore, facilitating optimal recovery of voluntary UE movement is a major concern in inpatient rehabilitation. If patients do not show recovery potential, therapists may shift to compensatory strategies to help patients regain their functions in daily activities. Therefore, accurate judgment of a patient’s voluntary UE movement recovery is an essential issue for rehabilitation therapists to provide proper interventions in patients with severe UE paresis.

The prediction of voluntary UE movement recovery in patients with severe UE paresis has not been examined well and remains inconclusive. Three reasons might explain such an observation. First, although previous studies have suggested that poor initial voluntary UE movement is associated with poor prognosis at or after discharge [6–9], these findings are difficult to generalize to patients with severe UE paresis. Previous studies investigated the association between initial UE impairment severity and recovery in a group of patients with heterogeneous severity of UE paresis [6–8]. However, the association estimated in a heterogeneous group is a weighted result among the pooled sample and may not represent all subgroups accurately, for different recovery patterns exist between different subgroups [10]. In other words, the estimated association between initial severe UE paresis with poor recovery may be biased when such results are analyzed together with mildly and moderately impaired patients. Therefore, further investigation with a homogeneous group of severe UE paresis patients is necessary.

Second, the outcome measures did not assess voluntary UE movement in most studies that investigated motor recovery in patients with severe paresis. For example, the Scandinavian Stroke Scale assesses muscle strength, which cannot describe whether an individual can perform isolated wrist or forearm movements [2]. Some studies used functional assessments of UE [11,12]. UE function is a broad term, covering a range of abilities including voluntary UE movement, muscle tone, multiple joints movement coordination, and adjusting interactions with objects [4]. For instance, “take up and put down an object” is a UE function that involves several joints in voluntary movements simultaneously; i.e., the thumb, fingers, elbow or shoulder. Different sizes and weights of objects also influence the results of functional assessments. Thus, the results of these studies are difficult to apply to the interpretation of voluntary UE movement recovery in patients with severe paresis. Voluntary UE movement is the foundation of UE function and reflects the basic control of the brain over the musculoskeletal system. Investigating voluntary UE movement recovery in patients with severe UE paresis provides the most fundamental research evidence regarding UE motor recovery after stroke.

Third, potential predictors have not been broadly explored in patients with severe UE paresis [5,8,13,14]. Initial severity of UE movement [5,13,14] and lesion locations [15] were associated with voluntary UE movement recovery at 3 or 6 months after stroke in patients with severe UE paresis. However, the initial severity of UE movement alone can explain only 16.0% of the variance of patients’ recovery [14]. It is unknown whether the other variables, such as duration after stroke onset and lesion volume, could be predictors as well and might increase the total predictive power for patients’ recovery. Therefore, we aimed to investigate the extent and variation of voluntary UE movement recovery during inpatient rehabilitation in patients with severe UE paresis. Furthermore, we aimed to investigate the best predictive model (i.e., minimal variables with maximal predictive power) of the recovery of voluntary UE movement by clinical variables in patients with severe UE paresis.

## Method

### Subjects

This was a single-center, prospective, observational study. The patients were consecutively recruited from a local medical center, from January 2009 to January 2012. The inclusion criteria for recruitment were (1) first-ever intracerebral hemorrhage or ischemic stroke, confirmed by either computed tomography (CT) or magnetic resonance imaging (MRI); (2) ability to communicate and follow 1-step instructions; (3) patients with severe UE paresis (i.e., the UE subscale of the Stroke Rehabilitation Assessment of Movement measure (STREAM-UE) [16] score at admission < = 5) at admission to the rehabilitation ward.

STREAM-UE < = 5 described patients’ affected UE movement as having partial scapular, shoulder, or elbow movement, but unable to reach the mouth level. Therefore, 5 was set as the cut-off point for defining patients with severe UE paresis (i.e., no or very limited voluntary UE movement). At the medical center, the inpatient rehabilitation program starts when patients are in a stable neurological condition and are transferred to rehabilitation ward, usually 2–3 weeks after stroke onset. All patients with severe UE paresis were transferred to inpatient rehabilitation for further treatment. Before being transferred to the rehabilitation ward, all patients received early bed-side rehabilitation, such as bed mobility or passive limb motor exercises, during their early care in the neurology or internal medical department.

All patients who were admitted to the inpatient rehabilitation ward had to participate in the inpatient rehabilitation program at the medical center. The length of stay in the program was up to 6 weeks, with an average of 3 to 4 weeks. The inpatient rehabilitation program was a multiple disciplinary program, including occupational therapy, physical therapy, and speech therapy where necessary; they received 30 minutes of each therapy per day, 5 times per week. The main therapy focus of the inpatient rehabilitation program was to improve the recovery of impaired body functions (e.g., voluntary movements, balance, cognition, etc.) and patients’ basic daily functions (e.g. eating, dressing, hygiene etc.). Patients were prepared for discharge either to home or to other institutes. Selection of either a remedial or a compensatory approach was highly dependent on individual therapists’ judgments. Patients were discharged when physicians and therapists agreed that the patients’ treatment goals had been achieved.

The participants were excluded if they (1) suffered from other central/ peripheral neurologic diseases, such as brain tumor or Parkinson’s disease, which could influence their motor control before or during recruitment; (2) stayed in inpatient rehabilitation for less than 7 days; (3) did not give informed consent. The reasons for the participants staying less than 7 days were mainly due to transfer to other wards or hospitals because of either changes in the patient’s condition or a desire on the part of the patient. They may not receive sufficient rehabilitation and, were therefore excluded to avoid bias in the results. This study was approved by the Research Ethics Committee Office of National Taiwan University Hospital. Written informed consent was obtained from each participant after screening for eligibility.

### Procedure

A trained research assistant screened the eligibility of each patient who was admitted to the rehabilitation ward. Eligible patients were then assessed for their baseline voluntary UE movement ability at admission within 7 days and assessed again within 3 days before discharge by the same research assistant. The patients’ demographic data, National Institutes of Health Stroke Scale (NIHSS) scores [17], and comorbidities from medical records were recorded by the research assistant. The recorded comorbidities included hypertension, diabetes mellitus, hyperlipidemia, and cardiac disease. Except for the assessment, the research assistant did not have any other contact with the patients that might bias the assessment results during the patients’ hospital stays. Neuroimaging data of the eligible patients were extracted by an independent radiologist using all of the patients’ brain CT or MRI images that were taken during their emergency and acute stage care.

### Outcome: Recovery of voluntary UE movement

Voluntary UE movement was assessed with the STREAM-UE [16] (see S1 Appendix). The STREAM-UE contains 10 items evaluating voluntary movement of UE segments. Each item is graded on a 3-point scale (0-1-2). A higher score represents better UE movement. The total possible score of the STREAM-UE ranges from 0 to 20 points. The STREAM-UE is a fast, easy to use, and standardized outcome measure [18,19]. An assessor is able to finish the assessment in 5 to 10 minutes. In addition, the STREAM-UE has been shown to have good psychometric properties, including reliability, validity, and responsiveness, in stroke patients [19–22]. Thus, the STREAM-UE is recommended in stroke-related clinical practice and research [18,23].

The participants’ STREAM-UE scores at discharge (DC_STREAM-UE_) were defined as the primary outcome measure for voluntary UE movement recovery. In addition to the DC_STREAM-UE_, which represents a patient’s absolute voluntary UE movement ability, we conducted a secondary outcome measure (i.e., Δ_STREAM-UE_) to represent the difference in magnitude of a patient’s voluntary UE movement during the inpatient rehabilitation stay. The Δ_STREAM-UE_ was calculated as the STREAM-UE scores at discharge minus the scores at admission. Because recovery is a process of change, the Δ_STREAM-UE_ may reflect the underlying biological change that influences patients’ recovery.

### Candidate predictors

Nineteen clinical variables (candidate predictors) were investigated. The candidate predictors included 3 categories: demographic data, neuroimaging data, and baseline severity of stroke. The demographic data included age, gender, time from onset to baseline assessment, time from baseline to final assessment, and history (or absence) of craniotomy.

The neuroimaging data included type of stroke, lesion location (6 categories), lesion volume, lesion volume with surrounding edema, and greatest midline shift. The 6 lesion location categories were: (1) mixed cortical and subcortical; (2) cortical including primary motor cortex; (3) cortical excluding primary motor cortex; (4) subcortical including basal ganglia; (5) subcortical excluding basal ganglia; and (6) brain stem. Patients’ lesions were first identified as involving certain anatomical structures: cortical area (i.e., frontal, temporal, parietal and occipital lobes), subcortical gray matter (i.e., basal ganglia, thalamus, mid-brain, pons, and medulla oblongata), and subcortical white matter (i.e., coronal radiate, and internal capsule). Second, according to the involved structures, each patient was assigned to one of the 6 categories. If a patient’s lesion extended from the cortex into the subcortical areas, he/she was categorized as category 1. If a patient had only cortical insult, the patient was categorized as category 2 or 3, according to whether the primary motor cortex was injured or not. Similarly, patients with only subcortical insult were categorized as 4 or 5, depending on whether the basal ganglia was insulted. Patients with lesions involved in the mid-brain, pons, or medulla oblongata were categorized as category 6.

Lesion volumes of infarction and hematoma were measured using the ABC/2 method [24]. A, B, and C represent the three perpendicular axes of lesions/hematomas, respectively. The ABC/2 method is accurate for measuring lesions with regular margins. Although lesion volume is slightly overestimated by the ABC/2, there is no significant difference between the ABC/2 and 3D measurements [25,26]. The ABC/2 has been shown to have excellent intrarater and interrater reliability [25,27]. If edema was present, a further index (i.e., volume of lesion and edema) was calculated for the volume of lesion plus the edema area surrounding the lesion, using the ABC/2 method. All neuroimaging data were extracted from patients’ medical imaging records by an independent radiologist with 13 years of clinical experience. Before formal patient recruitment, images of 10 patients with stroke were read by the radiologist and the results were confirmed by one of the authors (JSJ).

The baseline severity of stroke included 4 variables. The first was the baseline severity of voluntary UE movement, which was assessed with the STREAM-UE at admission to rehabilitation ward. The second was the baseline severity of stroke neurological deficits, assessed with the NIHSS. Higher scores of the NIHSS represent more severe stroke deficits [17]. The third and the fourth were shoulder abduction and finger extension ability, respectively, measured with items of the STREAM-UE. A recent study found that shoulder abduction and finger extension were early predictors of functional recovery of the affected UE [28].

### Data analysis

Descriptive statistics were used to describe the magnitude and the distribution of the outcome measures (i.e., DC_STREAM-UE_ and Δ_STREAM-UE_). We defined large improvement (Δ_STREAM-UE_ > = 10) as a change exceeding half of the STREAM-UE scale’s score range.

We first tried to find the best predictive model for predicting the DC_STREAM-UE_. We evaluated the associations between the 19 clinical variables and the DC_STREAM-UE_ with the Pearson correlation coefficient and the independent Student t test. The clinical variables with significance level at p < 0.1 in univariate analysis were considered as potential predictors and then entered into the multiple regression analyses. We chose p < 0.1 as the variable selection criterion to avoid the exclusion of possible important factors [29,30]. We used a stepwise selection method to build the final predictive model for the DC_STREAM-UE_. A variable had to be significant at a p value of 0.1 to be entered in the stepwise regression model, while a variable in the model had to be significant at the 0.05 level for it to remain in the model. A 2-tailed p value <0.05 was considered statistically significant. Multicollinearity among the predictors was examined by the variance inflation factor (VIF) and indicated by a VIF value >10. Second, the same approach was used for the Δ_STREAM-UE_ to find the best predictive model. Data were analyzed using R version 2.14 software [31].

## Results

**Fig 1:**
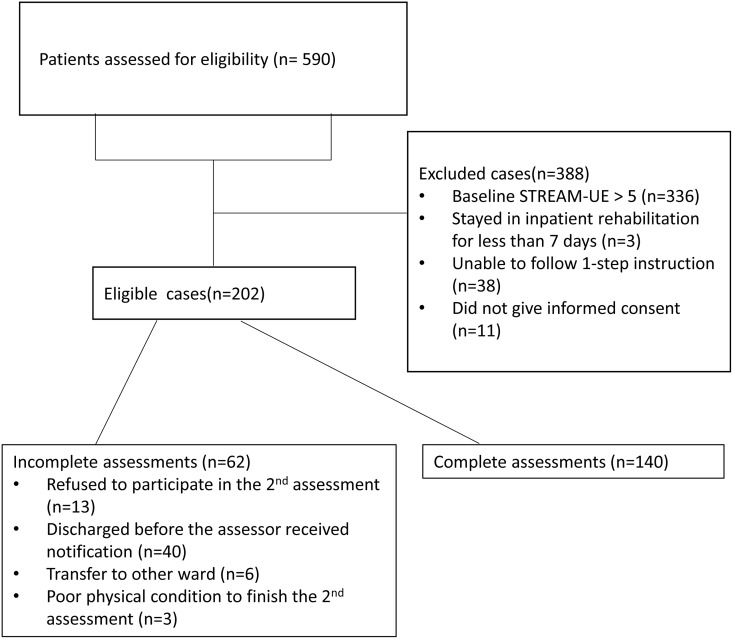
The patient enrolment flow diagram.

A total of 590 patients with stroke who were admitted to the rehabilitation ward were screened for eligibility. The process of patient recruitment is shown in the patient enrolment flow diagram (Fig 1). Of these, 202 eligible participants (34.2%) were enrolled in the study. Among the 202 enrolled participants, 62 participants (30.7%) did not complete the STREAM-UE assessment either at admission or at discharge and thus were excluded from further analysis. These 62 participants were not different from the rest of the participants who completed the assessments in terms of baseline STREAM-UE score (p = 0.161), age (p = 0.167), time from onset to baseline assessment (p = 0.416), time from baseline to final assessment (p = 0.353), or volume of lesion (p = 0.303). A total of 140 (68.3%) participants who completed both UE motor assessments remained for further analysis (mean age = 63.9 years; 63.6% male; 66.4% infarction). The characteristics of the participants are presented in Table 1.

**Table 1:** Demographics of the Study Patients.

	Infarct	Hemorrhage	Total
	(n = 93)	(n = 47)	(n = 140)
Male, n (%)	55 (59.1)	34 (72.3)	89 (63.6)
Age, year			
Mean (SD)	66.4 (12.0)	58.9 (12.6)	63.9 (12.7)
Range	30.4–88.1	28.5–82.7	28.5–88.1
Hypertension, n (%)	67 (72.0)	39 (83.0)	106 (75.7)
Hyperlipidemia, n (%)	44 (47.3)	11 (23.4)	55 (39.3)
Diabetes, n (%)	32 (34.4)	10 (21.3)	42 (30.0)
Cardiac disease, n (%)	47 (50.5)	6 (12.8)	53 (37.9)
Current smoking, n (%)	21 (22.6)	10 (21.3)	31 (22.1)
Alcohol drinking habit, n (%)	22 (23.7)	13 (27.7)	35 (25.0)
Time from onset to baseline, day			
Mean (SD)	23.3 (13.3)	22.1 (10.1)	22.9 (12.2)
Range	7–102	8–59	7–102
Time from baseline to discharge, day			
Mean (SD)	35.7 (43.8)	41.4 (13.1)	40.3 (14.4)
Range	9–74	13–91	9–91
Had received craniotomy, n (%)	6 (6.5)	5 (10.6)	11 (7.9)
Lesion location, n (%)			
Both cortical and subcortical	19 (20.4)	5 (10.6)	24 (17.1)
Cortical including primary motor cortex	18 (19.4)	5 (10.6)	23 (16.4)
Cortical excluding primary motor cortex	10 (10.8)	3 (6.4)	13 (9.3)
Subcortical including basal ganglia	12 (12.9)	22 (46.8)	34 (24.3)
Subcortical excluding basal ganglia	13 (14.0)	0 (0.0)	13 (9.3)
Brain stem	14 (15.1)	1 (2.1)	15 (10.7)
Lesion volume, mL			
Mean (SD)	92.2 (129.9)	37.5 (31.7)	75.4 (112.2)
Range	0.3–553.0	1.0–105.7	0.3–553.0
Lesion volume with surrounding edema, mL			
Mean (SD)	87.2 (128.0)	58.0 (59.4)	77.4 (110.5)
Range	0.3–553.0	5.7–263.8	0.3–553.0
Greatest midline shift, mm			
Mean (SD)	1.3 (3.7)	2.4 (3.0)	1.6 (3.5)
Range	0–19	0–9	0–19
STREAM-UE			
Baseline score			
Mean (SD)	1.0 (1.5)	0.9 (1.3)	1.0 (1.4)
Range	0.0–5.0	0.0–5.0	0.0–5.0
Discharge score			
Mean (SD)	4.2 (4.8)	6.8 (5.6)	5.1 (5.2)
Range	0.0–20.0	0.0–20.0	0.0–20.0
Change of score			
Mean (SD)	3.3 (4.2)	5.9 (5.3)	4.2 (4.7)
Range	-2.0–16.0	-1.0–17.5	-2.0–17.5

### The recovery of voluntary UE movement

The DC_STREAM-UE_ of the participants covered all ranges (0–20) of the STREAM-UE scale, with mean = 5.1 (SD = 5.2). Five participants (3.6%) achieved DC_STREAM-UE_ scores of higher than 15; 20 participants (14.3%) had scores above 10 and less than or equal to 15; 30 (21.4%) had scores above 5 and less than or equal to 10; 85 (60.7%) of the participants remained at < = 5.

The magnitude of Δ_STREAM-UE_ ranged from -2 to 17.5, mean (SD) = 4.2 (4.7). Twenty-three participants (16.4% of all participants) showed a large improvement (Δ_STREAM-UE_ > = 10). Twenty-three (16.4% of all participants) had a Δ_STREAM-UE_ of 6 to 9, while 48 (34.3% of all participants) had a Δ_STREAM-UE_ of 1 to 5. The rest of the participants (32.9% of all participants) had a Δ_STREAM-UE_ = 0 (n = 42) or a Δ_STREAM-UE_ < 0 (n = 4).

### Univariate associations between candidate predictors and outcome

**Fig 2:**
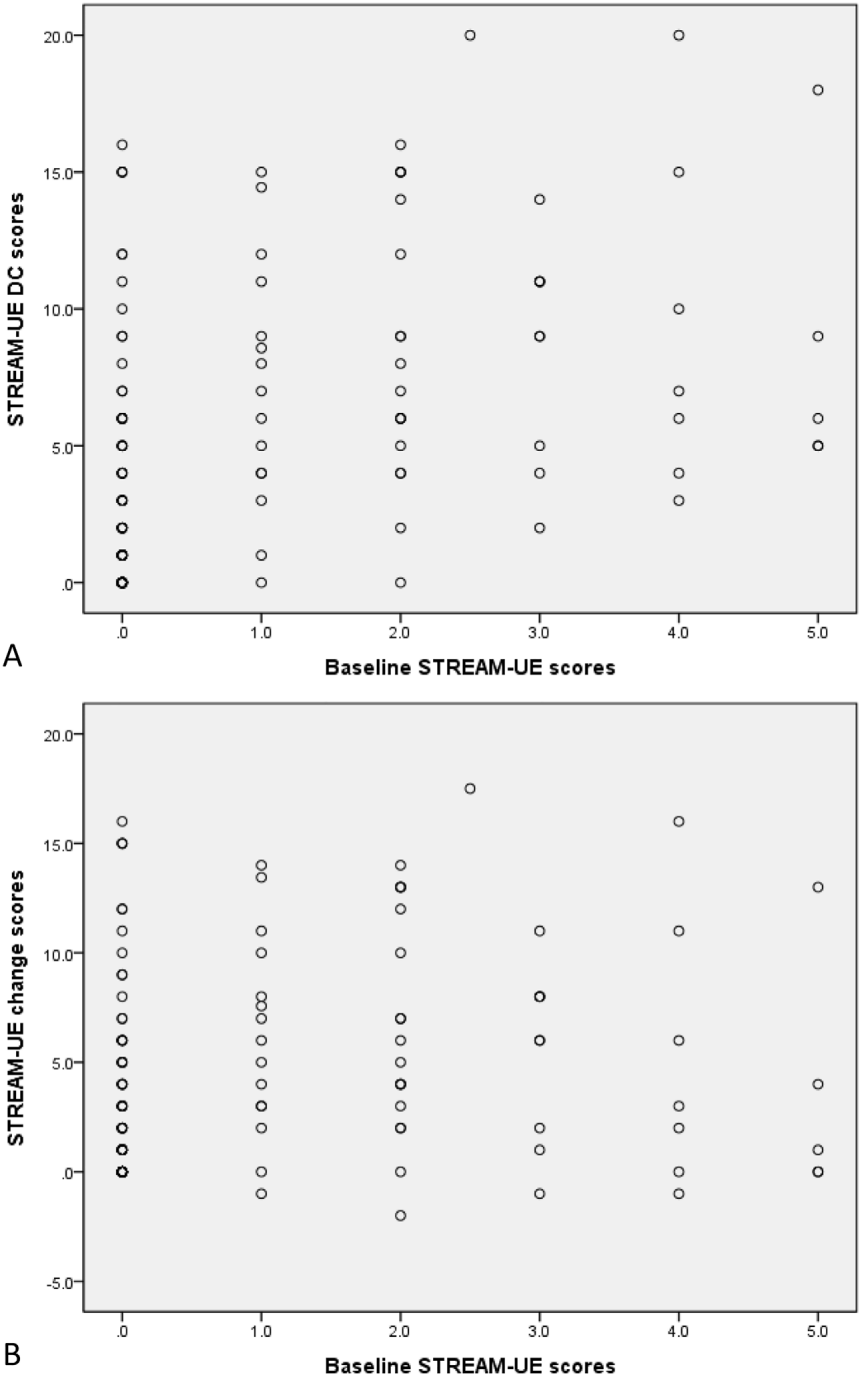
The scatter plots of baseline STREAM-UE scores versus STREAM-UE DC scores (A) and STREAM-UE change scores (B). STREAM-UE = the UE subscale of the Stroke Rehabilitation Assessment of Movement measure. DC = discharge.

The associations between each candidate predictor and the DC_STREAM-UE_ or the Δ_STREAM-UE_ are shown in Tables 2 and 3, respectively. Fig 2 shows the associations between baseline STREAM-UE scores and the two outcome measures. Ten predictors were selected for multiple regression analysis of the DC_STREAM-UE_ model, including stroke type, mixed cortical and subcortical lesion, cortical lesion excluding the primary motor cortex, lesion volume, lesion volume with surrounding edema, greatest midline shift, baseline STREAM-UE, NIHSS, shoulder abduction, and finger extension scores. Except for the greatest midline shift and baseline shoulder abduction scores (p > 0.1), the remaining 8 predictors were selected for the Δ_STREAM-UE_ model.

**Table 2:** Univariate analysis for the STREAM-UE scores at discharge (DC_STREAM-UE_) (n = 140).

	Univariate analysis		
	Pearson r (95% CI)	Student t (95% CI)	p
Gender		-0.18 (-2.05 ~ 1.71)	0.859
Age	-0.06 (-0.22 ~ 0.11)		0.503
Time from onset to baseline	-0.05 (-0.22 ~ 0.11)		0.530
Time from baseline to discharge	-0.07 (-0.14 ~ 0.19)		0.385
Had received craniotomy		-0.56 (-5.93 ~ 3.51)	0.584
Stroke type (hemorrhage v.s. infarct)		-2.67 (-4.47 ~ -0.65)	0.009*
Mixed cortical and subcortical lesion		4.61 (2.15 ~ 5.45)	< 0.001*
Cortical lesion including primary motor cortex		0.75 (-1.75 ~ 3.78)	0.459
Cortical lesion excluding primary motor cortex		-2.43 (-9.07 ~ -0.53)	0.030*
Subcortical lesion including basal ganglia		-0.41 (-2.36 ~ 1.56)	0.683
Subcortical lesion excluding basal ganglia		-0.66 (-2.30 ~ 1.18)	0.513
Brain stem lesion		-1.65 (-4.65 ~ 0.56)	0.116
Lesion volume	-0.30 (-0.45 ~ -0.13)		0.001*
Lesion volume with surrounding edema	-0.25 (-0.40 ~ -0.09)		0.003*
Greatest midline shift	-0.15 (-0.32 ~ 0.02)		0.086*
Baseline STREAM-UE score	0.47 (0.33 ~ 0.59)		< 0.001*
Baseline NIHSS score	-0.28 (-0.42 ~ -0.12)		0.001*
Baseline shoulder abduction^†^	0.23 (0.07 ~ 0.38)		0.007*
Baseline finger extension^†^	0.33 (0.17 ~ 0.47)		< 0.001*

STREAM = Stroke Rehabilitation Assessment of Movement.

NIHSS = National Institutes of Health Stroke Scale

* Variables with p value < 0.1 and were put in the regression model for selection

^†^ Shoulder abduction and finger extension scores were obtained from the STREAM-UE scale

**Table 3:** Univariate analysis for the STREAM-UE change scores (Δ_STREAM-UE_) (n = 140).

	Univariate analysis		
	Pearson r (95% CI)	Student t (95% CI)	p
Gender		-0.27 (-1.92 ~ 1.47)	0.791
Age	-0.08 (-0.24 ~ 0.09)		0.364
Time from onset to baseline	-0.11 (-0.27 ~ 0.06)		0.201
Time from baseline to discharge	0.01 (-0.12 ~ 0.21)		0.938
Had received craniotomy		-0.75 (-5.89 ~ 2.90)	0.470
Stroke type (hemorrhage v.s. infarct)		-2.91 (-4.33 ~ -0.81)	0.005*
Mixed cortical and subcortical lesion		3.43 (1.12 ~ 4.28)	0.001*
Cortical lesion including primary motor cortex		0.34 (-2.23 ~ 3.12)	0.736
Cortical lesion excluding primary motor cortex		-2.03 (-7.26 ~ -0.22)	0.063*
Subcortical lesion including basal ganglia		-0.47 (-2.21 ~ 1.37)	0.642
Subcortical lesion excluding basal ganglia		0.08(-2.07~2.24)	0.935
Brain stem lesion		-1.01 (-3.85~1.35)	0.327
Lesion volume	-0.27 (-0.42 ~ -0.10)		0.003*
Lesion volume with surrounding edema	-0.21 (-0.36 ~ -0.04)		0.015*
Greatest midline shift	-0.12 (-0.29 ~ 0.06)		0.183
Baseline STREAM-UE score	0.21 (0.05 ~ 0.37)		0.011*
Baseline NIHSS score	-0.22 (-0.37 ~ -0.06)		0.009*
Baseline shoulder abduction†	0.06 (-0.10 ~ 0.23)		0.460
Baseline finger extension†	0.24 (0.07 ~ 0.39)		0.005*

STREAM = Stroke Rehabilitation Assessment of Movement.

NIHSS = National Institutes of Health Stroke Scale

* Variables with p value < 0.1 and were put in the regression model for selection

^†^ Shoulder abduction and finger extension scores were obtained from the STREAM-UE scale

### Multivariate associations between candidate predictors and outcome measures

The results of the stepwise multiple regression analyses are presented in Table 4. For the DC_STREAM-UE_ model, baseline STREAM-UE score, hemorrhagic stroke, baseline NIHSS score, and cortical lesion excluding primary motor cortex were the 4 significant predictors (R^2^ = 0.35, p < 0.001). The baseline STREAM-UE score alone explains 19.0% of the variance of the DC_STREAM-UE_, while the remaining 3 predictors explained an additional 16.0% of that variance. The same 3 predictors, i.e., hemorrhagic stroke, baseline NIHSS score, and cortical lesion excluding the primary motor cortex, were found to be significant predictors of the Δ_STREAM-UE_. The 3 predictors together explained 22.0% of the variance in the Δ_STREAM-UE_ (p < 0.001). All VIF values were less than 1.2, indicating that multicollinearity among the predictors did not influence the regression estimates.

**Table 4:** Multiple regression analysis for the DC_STREAM-UE_ and Δ_STREAM-UE_ (n = 140).

		Unstandardized coefficients					Standardized coefficients	
Dependent variable	Independent variable	R	R^2^	R^2^ Change	B	95% CI for B	β	p
DC_STREAM-UE_	(Constant)	-	-	-	5.87	[3.55, 8.18]	-	< 0.001
	Baseline STREAM-UE score	0.43^a^	0.19	0.19	1.13	[0.54, 1.71]	0.3	< 0.001
	Hemorrhagic stroke	0.51^b^	0.26	0.07	3.88	[2.15, 5.60]	0.34	< 0.001
	Baseline NIHSS score	0.57^c^	0.32	0.06	-0.24	[-0.39, -0.09]	-0.25	0.002
	Cortical, primary motor cortex uninvolved	0.59^d^	0.35	0.03	3.14	[0.50, 5.77]	0.18	0.02
Δ_STREAM-UE_	(Constant)	-	-	-	6.05	[3.98, 8.12]	-	< 0.001
	Hemorrhagic stroke	0.30^e^	0.09	0.09	3.83	[2.15, 5.50]	0.37	< 0.001
	Baseline NIHSS score	0.42^f^	0.18	0.09	-0.25	[-0.39, -0.11]	-0.28	0.001
	Cortical, primary motor cortex uninvolved	0.47^g^	0.22	0.04	3.33	[0.83, 5.84]	0.21	0.009

ΔSTREAM-UE = change score of STREAM-UE

^a.^ Predictors: (Constant), Baseline STREAM-UE score

^b.^ Predictors: (Constant), Baseline STREAM-UE score, Hemorrhagic stroke

^c.^ Predictors: (Constant), Baseline STREAM-UE score, Hemorrhagic stroke, Baseline NIHSS score

^d.^ Predictors: (Constant), Baseline STREAM-UE score, Hemorrhagic stroke, Baseline NIHSS score, Cortical-primary motor cortex uninvolved

^e.^ Predictors: (Constant), Hemorrhagic stroke

^f.^ Predictors: (Constant), Hemorrhagic stroke, Baseline NIHSS score

^g.^ Predictors: (Constant), Hemorrhagic stroke, Baseline NIHSS score, Cortical-primary motor cortex uninvolved

## Discussion

Using a relatively homogeneous cohort, we found wide variations in the recovery of voluntary UE movement in inpatients who suffered from severe UE paresis after stroke. About 16% of the participants had large improvements (Δ_STREAM-UE_ > = 10) and about 4% could achieve nearly full recovery at discharge (DC_STREAM-UE_ > 15). Most of the participants (67.1%) showed voluntary UE movement improvement. Our results indicate that large motor recovery remains possible even in patients with severe UE paresis. Previous studies have suggested the use of compensatory techniques and assistive devices for patients who show initially severe UE paresis because poor prognosis is expected [2,8]. However, we suggest exercising caution when planning treatment for this particular group of patients because substantial improvement has been demonstrated.

Three out of the 19 variables were selected as positive predictors of both outcome measures (i.e., DC_STREAM-UE_ and Δ_STREAM-UE_) in patients with severe paresis, which were hemorrhagic stroke, low NIHSS score at baseline, and cortical lesion excluding primary motor cortex. Shelton et al. found no significant difference among the effects of involvement of primary, premotor, or supplementary motor areas on UE motor recovery in patients with severe paresis [15]. The effect of subcortical lesion was not excluded in their analysis, thus this may have confounded their results and caused a discrepancy with our findings. Previous studies found hemorrhagic stroke and low initial NIHSS score were two predictors for good recovery in patients’ daily function [32,33]. Here, we have demonstrated that three variables are associated with UE movement recovery in patients with severe UE paresis. Because voluntary UE movement is the foundation for a paretic arm to regain function for daily activities, clinicians and researchers might consider hemorrhagic stroke, a low NIHSS score, and cortical lesion excluding the primary motor cortex as three important factors for selecting patients who may benefit more than other patients from remedial interventions.

We found that 35.0% and 22.0% of the variance of patients’ recovery could be explained by clinical variables in the DC_STREAM-UE_ and Δ_STREAM-UE_ models, respectively. For the DC_STREAM-UE_ model, only 16% variance of patients’ recovery could be explained by clinical variables after controlling the contribution of the baseline STREAM score. The results of both DC_STREAM-UE_ and Δ_STREAM-UE_ models indicate that the UE movement recovery of patients with severe UE paresis is predictive to a very limited degree by clinical variables. Zarahn’s study also found a poor prediction (R^2^ = 16%) of the voluntary UE movement recovery in patients with severe UE paresis [14]. The aforementioned wide variations of patients’ recovery indicates the complex nature of patients’ UE movement recovery. The large unexplained variance in recovery further indicates that there are unidentified biological mechanisms underpinning voluntary movement recovery after stroke. Therefore, clinicians and researchers should be cautious to avoid underestimating recovery potentials in patients with severe UE paresis. At present, accurate prediction of UE recovery in patients with severe UE paresis remains difficult. Further research is warranted in this particular group of patients to find key predictors and mechanisms for their UE movement recovery.

It may be argued that the limited predictive power of the selected clinical variables is due to a lack of some important variables. For example, neurophysiological (e.g., muscle motor-evoked potentials, MEPs) and neuroimaging (e.g. functional magnetic resonance and diffusion tensor imaging) measures have been suggested as predictors for motor recovery [14,34,35]. However, evidence of the predictive power of these measures for patients with severe UE paresis remains limited and controversial [5]. Furthermore, neurophysiological and neuroimaging measures are difficult to obtain in clinical settings because they require special equipment and complex analysis processes, and they are not suitable for all patients (e.g., patients with pacemakers or history of epilepsy). Developing an algorithm combining the use of clinical, neurophysiological and neuroimaging measures may help to increase the accuracy of prediction for stroke patients’ motor recovery [36]. However, further research for finding key predictors for patients with severe UE paresis remains warranted.

We investigated voluntary UE movement recovery using two outcome measures. First, the DC_STREAM-UE_, as an end-point measure, describes an individual’s ability for voluntary UE movement at discharge. This measure can help clinicians understand an individual’s final motor status and could help clinicians make proper arrangements for patients after their discharge. Second, the Δ_STREAM-UE_ represents the amount of change/improvement of an individual during their stay in a rehabilitation ward. The Δ_STREAM-UE_ may reflect the underlying changing process of neural substrates that are involved in voluntary UE movement [37,38]. Thus, for patients with severe UE paresis, the Δ_STREAM-UE_ implies the existence and the magnitude of the underlying neurological recovery that leads to permanent voluntary UE movement recovery. However, the floor and ceiling effects of a measure could impact observed change scores by reducing the detectability of a patient’s change. For those with severe UE paresis, we are concerned that the slight floor effect of the STREAM-UE at admission [19] might have reduced the strength of the associations between predictors and the Δ_STREAM-UE_, thus reducing the predictive power. Further studies could use outcome measures that are sensitive to motor change in patients with severe paresis in particular, such as electromyography, to increase the accuracy of prediction. Nonetheless, our investigation of the two outcome measures at the same time provides a comprehensive view of patients’ voluntary UE movement.

To generalize our results to other clinical settings may require careful consideration. In our setting, patients with stroke are transferred to inpatient rehabilitation wards after acute medical care (usually 3 to 7 days) has stabilized patients’ physical and neurological conditions. They then receive regular rehabilitation and may stay in the inpatient rehabilitation wards for an average of 1 month, which is considered a long length of stay. Given that our participants were mainly in the subacute stage, the results of the present study are hardly applicable to acute care wards. However, we did not find a significant association between the length of stay in a rehabilitation ward and voluntary UE movement recovery. Thus, our results may be generalized to other clinical settings for treating patients with different lengths of stay in rehabilitation wards. Nonetheless, further study is warranted to validate whether our findings are generalizable to patients treated in acute care settings or patients with short lengths of stay.

Five limitations of this study can be raised to narrow the interpretation of our findings. First, the time period of the study was focused only on inpatient rehabilitation. Our results cannot be generalized to long-term motor recovery. Second, we excluded patients who stayed in the rehabilitation ward for less than 7 days. These patients stayed for such short periods mainly due to transfers to other wards or hospitals because of changes in patients’ conditions or patients’ requests. We cannot rule out that some of these patients might have had fast recoveries and been discharged from the rehabilitation ward. Thus, excluding the patients with short stays might have caused unknown impacts on our results. Third, we did not measure the UE therapy dose. Although all participants received a regular 5-day rehabilitation program, the total amount of UE training might have varied among individuals. Fourth, although the ratio of the participants and the clinical variables was more than 10:1 in the present study, a larger sample size would allow for more accurate interpretations of the associations between clinical variables and voluntary UE movement recovery. Finally, our results are limited to generalization on functional performance of UE (e.g., dressing and grasping a glass). Further research is warranted to investigate functional recovery of UE and its predictors in patients with severe UE paresis to extend our findings.

## Conclusion

Using a relatively homogeneous cohort, we found that the recovery of voluntary UE movement varied (from no improvement to fully recovery) in patients with severe UE paresis who received regular inpatient rehabilitation training. Hemorrhagic stroke, baseline NIHSS score, and cortical lesion excluding primary motor cortex were the three clinical variables associated with the recovery of voluntary UE movement in patients with severe UE paresis. Baseline STREAM-UE scores were associated with the DC_STREAM-UE_ but not the Δ_STREAM-UE_. Nonetheless, the predictive power of the clinical variables were poor in predicting the DC_STREAM-UE_ and the Δ_STREAM-UE_. These results indicate the complex nature of motor recovery in patients with severe UE paresis and that it is difficult to make accurate predictions by the currently used clinical variables. Future research is needed to investigate clinical variables other than those in the present study.

## Supporting Information

S1 Appendix (DOCX)
