# Supplementary material for: Predicting Recovery of Voluntary Upper Extremity Movement in Subacute Stroke Patients with Severe Upper Extremity Paresis
Source: PLoS One. 2015 May 14;10(5):e0126857. doi: 10.1371/journal.pone.0126857 (PMC4431803; doi:10.1371/journal.pone.0126857)
Supplement: S1 Appendix — (DOCX) [file pone.0126857.s001.docx]

Appendix 1. Items and the scoring criteria of the UE subscale of the Stroke Rehabilitation Assessment of Movement measure.

| Item number | Test movement | Scoring |
| --- | --- | --- |
| 1 | Protracts scapular in supine | 0 – unable to perform the test movement through any appreciable range  1 – able to perform only part of the test movement or able to complete the movement, but only with marked deviation from normal pattern  2 – able to complete the movement in a manner that is comparable to the unaffected side |
| 2 | Extends elbow in supine |  |
| 3 | Shrugs shoulders |  |
| 4 | Raises hand to touch top of head |  |
| 5 | Hand to sacrum |  |
| 6 | Raises arm overhead to fullest elevation |  |
| 7 | Supinates and pronates forearm |  |
| 8 | Closes hand from fully opened position |  |
| 9 | Opens hand from fully closed position |  |
| 10 | Opposes thumb to index finger (tip to tip) |  |
